# *PRKCDBP* Methylation is a Potential and Promising Candidate Biomarker for Non-small Cell Lung Cancer

**DOI:** 10.3779/j.issn.1009-3419.2022.102.03

**Published:** 2022-02-20

**Authors:** Jing LI, Lin QI, Mingfang ZHANG, Caiyun YAO, Jinan FENG, Zhonghua ZHENG, Chujia CHEN, Shiwei DUAN, Yuanlin QI

**Affiliations:** 1 School of Basic Medical Sciences, Fujian Medical University, Fuzhou 350122, China; 2 Medical Genetics Center, School of Medicine, Ningbo University, Ningbo 315211, China

**Keywords:** Lung neoplasms, PRKCDBP, DNA methylation

## Abstract

**Background and objectives:**

The occurrence and development of lung cancer are closely linked to epigenetic modification. Abnormal DNA methylation in the CpG island region of genes has been found in many cancers. Protein kinase C delta binding protein (PRKCDBP) is a potential tumor suppressor and its epigenetic changes are found in many human malignancies. This study investigated the possibility of *PRKCDBP* methylation as a potential biomarker for non-small cell lung cancer (NSCLC).

**Methods:**

We measured the methylation levels of *PRKCDBP* in the three groups of NSCLC tissues. Promoter activity was measured by the dual luciferase assay, with 5'-aza-deoxycytidine to examine the effect of demethylation on the expression level of PRKCDBP.

**Results:**

The methylation levels of *PRKCDBP* in tumor tissues and 3 cm para-tumor were higher than those of distant (> 10 cm) non-tumor tissues. Receiver operating characteristic (ROC) curve analysis between tumor tissues and distant non-tumor tissues showed that the area under the line (AUC) was 0.717. Dual luciferase experiment confirmed that the promoter region was able to promote gene expression. Meanwhile, *in vitro* methylation of the fragment (*PRKCDBP_Me*) could significantly reduce the promoter activity of the fragment. Demethylation of 5'-aza-deoxycytidine in lung cancer cell lines A549 and H1299 showed a significant up-regulation of PRKCDBP mRNA levels.

**Conclusion:**

*PRKCDBP* methylation is a potential and promising candidate biomarker for non-small cell lung cancer.

## Introduction

The latest data show that one in four cancer patients in the United States is lung cancer^[1]^, and it is also the highest in cancer mortality in China^[2]^. Clinically, 80%-85% of lung cancer patients are non-small cell lung cancer (NSCLC)^[3]^. The survival rate of lung cancer depends largely on the stage of diagnosis. The five-year survival rate of lung cancer diagnosed at local stage is 54%, but only 15% of patients can be diagnosed at local stage^[4]^. At present, clinical diagnosis of lung cancer relies on spiral computed tomography (CT) and X-ray, but it is prone to both false negative and false positive cases^[5]^. Therefore, finding new diagnostic markers for lung cancer is of great significance for lung cancer.

The occurrence and development of lung cancer is closely linked to epigenetic modification^[6]^. DNA methylation is one of the most studied epigenetics, and abnormal DNA methylation in the CpG island region of genes has been found in many cancers^[7]^. CpG island mainly occurs in the coding region of eukaryotic genes, and abnormal methylation of CpG island will cause down-regulation of tumor suppressor gene expression or overexpression of oncogene, thereby promoting the development of cancer^[8]^.

The protein kinase C delta binding protein (*PRKCDBP*) is a potential tumor suppressor and its epigenetic changes are found in many human malignancies. The *PRKCDBP* gene is located at the human chromosome 11p15.5-p15.4, which harbors frequent deletions in the tumors of breast cancer^[9]^ and lung cancer^[10]^, suggesting its possible role in tumorigenesis. *PRKCDBP* can induce G_1_ cell arrest, enhance the sensitivity of cells to various apoptotic stimuli, and promote apoptosis. In addition, *PRKCDBP* transcription is found to be directly activated by tumor necrosis factor α (TNFα) via nuclear factor kappa-B (NF-κB) signaling pathway^[11]^. TNFα is an important pro-inflammatory cytokine that not only plays an important role in the inflammatory process, but also involves processes such as apoptosis, proliferation, differentiation, wich are often related to the induction and development of cancer^[12]^. As a transcriptional target of the TNFα-NF-κB signaling pathway, *PRKCDBP* plays a key role in TNFα-induced apoptosis^[11]^.

To explore whether *PRKCDBP* can be used as a diagnostic biomarker for NSCLC. This experiment detected the level of *PRKCDBP* methylation in tumor tissues, 3 cm para-tumor tissues, distant non-tumor from NSCLC patients. We conducted an in-depth analysis of the *PRKCDBP* methylation level and clinical pathological data, and verified the gene regulation function of the target gene fragment.

## Materials and methods

### Collection of tissue samples

We have collected tissues samples of NSCLC patients under the guidance of pathologists since February 2018. The tissue samples comprised tumor tissues, para-tumor tissues (3 cm away from tumor tissue) and distant non-tumor tissues (more than 10 cm from tumor tissues). We collected a total of 119 samples, including 44 pairs of tumor tissues and distant non-tumor tissues, and 31 para-tumor tissues. We collected clinical pathology information for NSCLC patients, including age, gender, family history, and pathological type. The study protocol was approved by the Ethics Committee of the First Affiliated Hospital of Fujian Medical University, and each patient signed an informed consent form.

### Bisulfite conversion

DNA of tissue samples were extracted using EZNA Tissue DNA Extraction Kit (Omega Bio-Tek, Norcross, GA, USA) and quantified using a NanoDrop 1000 spectrophotometer (Thermal Scientific Co. Ltd., Wilmington, MA, USA). The bisulfite modification was performed according to the instruction of EZ DNA Methylation-Gold Kit (Zymo Research, Orange, CA, USA). The converted DNA was stored at -20 ℃ for methylation detection.

### Quantitative methylation specific PCR (qMSP)

The qMSP primers were designed to amplify the CpG rich region of *PRKCDBP* gene, and the qMSP product was 96 bp in length. The forward primer was 5'-ATA GGTCGGTAAAGGTTT-3', and the reverse primer was 5'-CCACGAACTACTAATAACG-3'. We used a SYBR-green-based qMSP to assess the level of methylation of *PRKCDBP* in three groups of NSCLC tissue samples. In order to avoid errors in the amount of sample loading, each sample used ACTB as an internal control. The primer sequences of ACTB were as follows: Forward, 5'-TGGTGATGGAGGAGGTTTAGTAAGT-3' and reverse, 5'-AACCAATAAAACCTACTCCTCCCTTAA-3'. At the same time, M-SssI-treated human sperm DNA and nuclease-free water were used as positive and negative controls, respectively, to ensure the accuracy of the experiment. The qMSP product was randomly selected for capillary electrophoresis (Bioptic, Taiwan, China) and Sanger sequencing to verify the homogeneity of product and the successful conversion of sodium bisulfite. We used the formula to calculate the methylation reference percentage (PMR) value. The relevant formula was as follows: [PMR=2^-ΔΔCt^×100%, ΔΔCt=sample DNA (Ct_PRKCDBP_-Ct_ACTB_)-fully methylated DNA (Ct_PRKCDBP_-Ct_ACTB_)].

### Dual luciferase and real-time PCR for gene expression detection

The PKCDBP CpG-rich fragment (+181 bp to +581 bp) was selected to construct the pGL3-*PRKCDBP* plasmid of the target gene. To avoid the difference in the number of cells, the pGL3-basic plasmid and the pRL-SV40 plasmid were co-transformed as a negative control, and the pGL3-promoter plasmid and the pRL-SV40 plasmid were co-transformed as a positive control. Human embryonic kidney 293T cell line was used for the transformation. After 24 h of transfection, luciferase assays were performed using SpectraMax 190 (Molecular Devices, Sunnyv, USA) following the Promega Dual Luciferase Reporting System kit instructions. Luciferase activity was finally calculated using the Dual Luciferase Reporter Assay Systems (Promega, Madison City, WI, USA).

Lung cancer cell lines A549 and H1299 were purchased from the cell bank of Shanghai Academy of Sciences, and cells were treated with 5'-aza-deoxycytidine. RNA was extracted by TRIzol one-step method before and after treatment, and product cDNA was obtained using Takara reverse transcription kit. The forward RT-PCR primer for *PRKCDBP* was 5'-GCGGGAAGCTCCACGTTC-3', and the reverse primer was 5'-GCTCTGTACCTTCTGCAATCCG-3'. In order to avoid the error in the amount of sample loading, each sample had ACTB as the internal reference. The forward RT-PCR primer for ACTB was 5'-AGCACAGAGCCTCGCCTTT-3', and the reverse primer was 5'-AGGGTGAGGATGCCTCTCTT-3'. A negative reference was also made using nuclease-free water. The real-time PCR system was configured according to the Takara TB Green kit instructions, and the mRNA expression of *PRKCDBP* was detected using an StepOnePlus^TM^ (96) real-time PCR instrument (Applied Biosystems, Foster City, CA, USA).

### Bioinformatics analysis

*PRKCDBP* methylation levels of tumor tissuses and para-tumor tissues from 51 lung cancer patients were downloaded from TCGA database (https://genome-cancer.ucsc.edu/). In addition, we obtained data of 230 samples from the cBioPortal database (http://www.cbioportal.org) to analyze the relationship between *PRKCDBP* methylation level and mRNA expression.

### Statistical analysis

We performed statistical analysis using SPSS version 20.0 (SPSS Inc. Chicago, IL, USA). Nonparametric Friedman test was used to determine methylation differences between two groups among tumor tissues, 3-cm para-tumor tissues, and distant non-tumor tissues. Adjusted *P* was obtained by *Bonferroni's* correction. CpG site methylation of tumor tissues and para-tumor tissues from TCGA were compared using a *Wilcoxon signed-rank* test. *Kaplan-Meier* survival analysis was used to assess the prognostic value of the level of *PRKCDBP* methylation. ROC curve was used to evaluate the diagnostic value of distal *PRKCDBP* methylation for NSCLC. *P* < 0.05 was considered statistically significant.

## Results

### Descriptions of tissue samples and promoter region of PRKCDBP

We collected paired tissue samples from 44 patients with NSCLC and classified their clinical pathology information (Tab 1). The average age of the patients was 62.91±9.05 years old, including 23 females and 21 males. There were 32 cases of adenocarcinoma and 12 cases of squamous cell carcinoma.

**Table 1:** Clinical pathological information of NSCLC samples

Variables	Tumor PMR (%)	Para-tumor PMR (%)	Distant non-tumor PMR (%)	Tumor *vs* distant non-tumor			Para-tumor *vs* distant non-tumor	
				*P*	*P*_Adjusted_		*P*	*P*_Adjusted_
Age (yr)								
≤60 (*n*=16)	1.480 (0.420, 8.380)	1.400 (0.739, 3.390)	0.445 (0.040, 0.958)	0.003	0.008		0.024	0.073
> 60 (*n*=28)	1.390 (0.432, 5.407)	1.380 (0.415, 3.120)	0.394 (0.108, 1.640)	0.002	0.006		0.201	0.604
Gender								
Female (*n*=23)	0.726 (0.332, 2.580)	0.961 (0.513, 2.757)	0.200 (0.000, 3, 0.445)	0.001	0.002		0.007	0.022
Male (*n*=21)	1.605 (0.888, 94.555)	1.890 (0.501, 3.630)	1.310 (0.612, 2.010)	0.006	0.018		0.433	> 0.999
Smoking history								
Never-smoker (*n*=34)	1.460 (0.417, 4.735)	1.280 (0.499, 2.765)	0.276 (0.009, 0.890)	0.002	0.005		0.067	0.2
Smoker (*n*=10)	1.400 (0.387, 151.425)	3.140 (0.585, 16.650)	1.315 (0.573, 2.652)	0.002	0.005		0.114	0.342
Histological type								
LUAD (*n*=32)	1.030 (0.335, 4.735)	0.801 (0.302, 1.932)	0.295 (0.300, 0.950)	0.005	0.014		0.034	0.102
LUSC (*n*=12)	2.030 (1.237, 127.175)	2.970 (1.700, 3.470)	0.808 (0.042, 1.850)	0.001	0.004		0.186	0.558
Cancer location								
Right lung (*n*=26)	1.345 (0.067, 6.137)	1.935 (0.515, 3.300)	0.699 (0.006, 1.317)	0.002	0.007		0.131	0.392
Left lung (*n*=18)	1.460 (0.456, 7.220)	1.120 (0.498, 1.890)	0.276 (0.480, 0.913)	0.002	0.007		0.059	0.178
The data was represented by the median (quartile). The *P* value and the *P*_Adjusted_ value were calculated using a nonparametric *Friedman* test, and *P*_adjusted_ a Bonferroni adjusted *P* value. *P* < 0.05 was in bold font. PMR: methylation reference percentage; LUAD: lung adenocarcinoma; LUSC: Lung squamous cell carcinoma								

**Figure 1:**
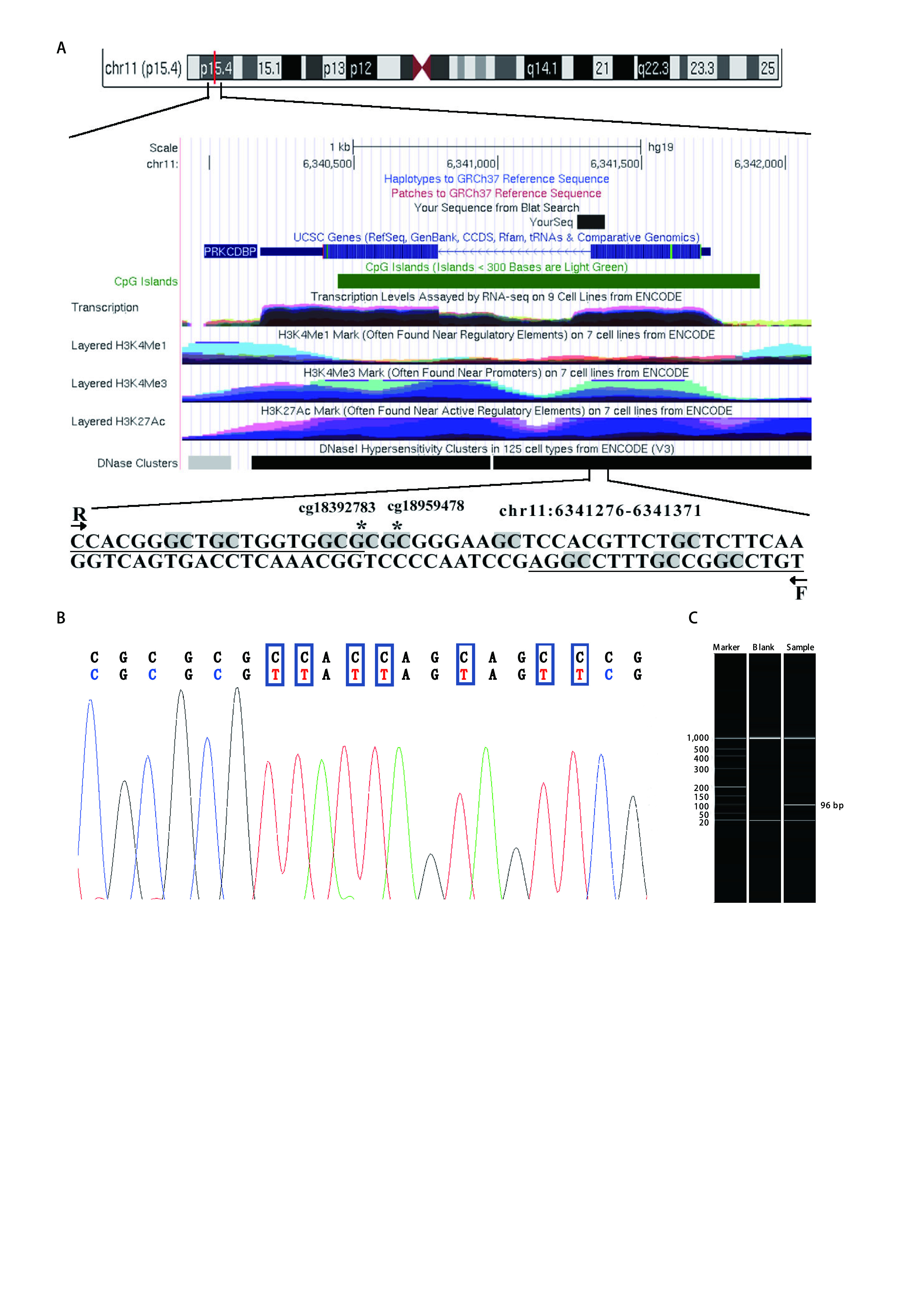
The qMSP primers and target sequences of the CpG island region of the *PRKCDBP* gene. A: F and R represented the forward and reverse primers, and all CG sites were grayed out. The asterisk CG site was the CG site screened by the TCGA database; B: The upper line was the original sequence, and the lower line was the converted sequence; C: Capillary gel electrophoresis verified that the size of amplified product was identical to the expected result. TCGA: The Cancer Genome Atlas.

Since the silencing of many tumor suppressor factors is due to the methylation of CpG islands, we selected a fragment in the CpG island region in the promoter region of *PRKCDBP* (GRCh37/hg19, chr6341276-chr66341371; Fig 1A). There are two CG sites (cg18959478 and cg18392783; see also Fig 4C) in the selected fragment (Fig 1A). Capillary electrophoresis showed the fragment size of qMSP product was the same as we expected (Fig 1C). The qMSP products were also subjected to Sanger sequencing and the results showed that the amplified sequences were correct, suggesting that the bisulfite conversion was complete (Fig 1B).

### Hypermethylation of PRKCDBP promoter in tumor tissues

**Figure 2:**
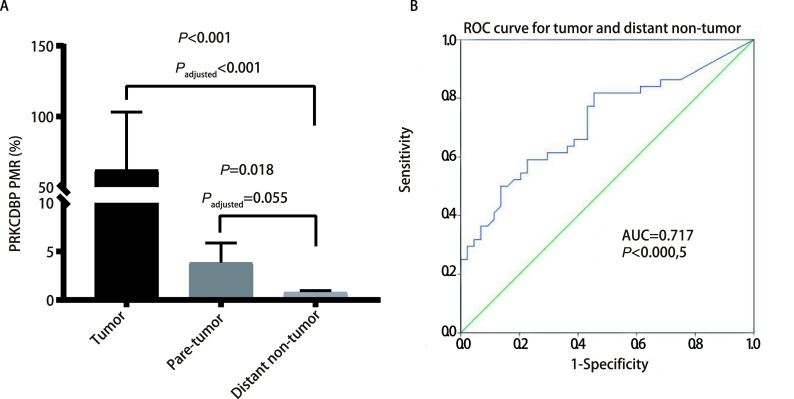
Differences in methylation levels between two groups and diagnostic value of *PRKCDBP* for lung cancer. A: Comparison of methylation levels of *PRKCDBP* in tumor tissues, 3 cm para-tumor tissues, and distant (over 10 cm) non-tumor tissues; B: The diagnostic value of *PRKCDBP* methylation for NSCLC. ROC: receiver operating characteristic; NSCLC: non-small cell lung cancer.

The comparison between groups in the three groups of samples was performed using the nonparametric Friedman test. *P*_adjusted_ is the Bonferroni corrected *P* value, which adjusts the type I error in multiple comparisons, and thus it is more accurate than the original *P* value. Paired comparison of *PRKCDBP* methylation levels showed that the methylation level of tumor tissues and 3 cm para-tumor tissues was higher than that of distant non-tumor tissues (tumor tissues *vs* distant non-tumor tissues: *P*_adjusted_ < 0.001; 3 cm para-tumor tissues *vs* distant non-tumor tissues: *P*_adjusted_=0.05; Fig 2A).

Through the TCGA database, we analyzed the methylation levels of four CpG sites located in the promoter region of *PRKCDBP*. The methylation levels of 3 CpG sites in tumor tissues was higher than those in matched para-tumor tissues (cg18959478, *P*=0.011; cg18392783, *P*=0.009; cg05628549, *P*=0.001, Fig 4C).

### Potential diagnosis and prognosis values of PRKCDBP methylation

Our ROC curve analysis showed that *PRKCDBP* methylation was of diagnostic value for NSCLC (AUC=0.717, *P*=0.000, 5, 95%CI: 0.609-0.824, sensitivity=59.09%, specificity=77.27%, Fig 2B). In addition, the ROC curve results of tumor tissues and para-tumor tissues in the TCGA database also showed that *PRKCDBP* methylation was a potential diagnostic biomarker for lung cancer (AUC=0.686, *P*=3E-6, 95%CI: 0.612-0.760, sensitivity=57.94%, Specificity=81.30%, Fig 4E). *Kaplan-Meier* survival analysis of TCGA data also indicated that overall survival was significantly lower in lung cancer patients with hypermethylation of *PRKCDBP* than in patients with hypomethylation of *PRKCDBP* (*P*=0.012, Fig 4D).

### Subgroup analysis of PRKCDBP methylation

Subgroup comparisons by age, gender, smoking history, lesion location and pathological type showed that the *PRKCDBP* methylation level in tumor tissues was higher than that in distant non-tumor tissues (all *P*_*adjusted*_ < 0.05, Tab 1). There was no significant difference in the methylation level between the tumor tissues and the 3 cm para-tumor tissues in the subgroup comparisons (all *P*_*adjusted*_ > 0.05). This may be due to the close proximity of the para-tumor tissues and tumor tissues.

### PRKCDBP mRNA expression is regulated by promoter methylation

**Figure 3:**
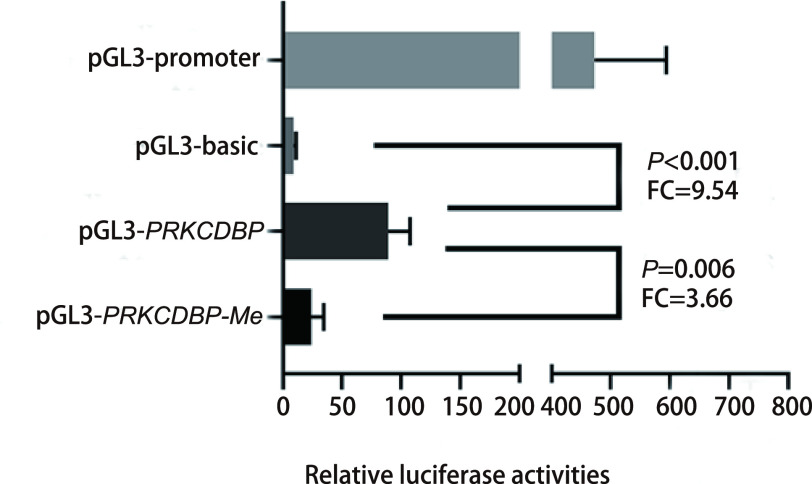
Dual luciferase reporter gene assay in human embryonic kidney 293T cells. The pGL3-promoter and pGL3-basic plasmids were positive and negative controls, respectively. *PRKCDB*: *PRKCDB* fragment (400 bp, chr11: 6319932-6320331) inserted in the multiple cloning region of pGL3-basic plasmid; *PRKCDBP_Me*: PRKCDBP plasmid under *in vitro* methylation treatment. FC: fold change.

**Figure 4:**
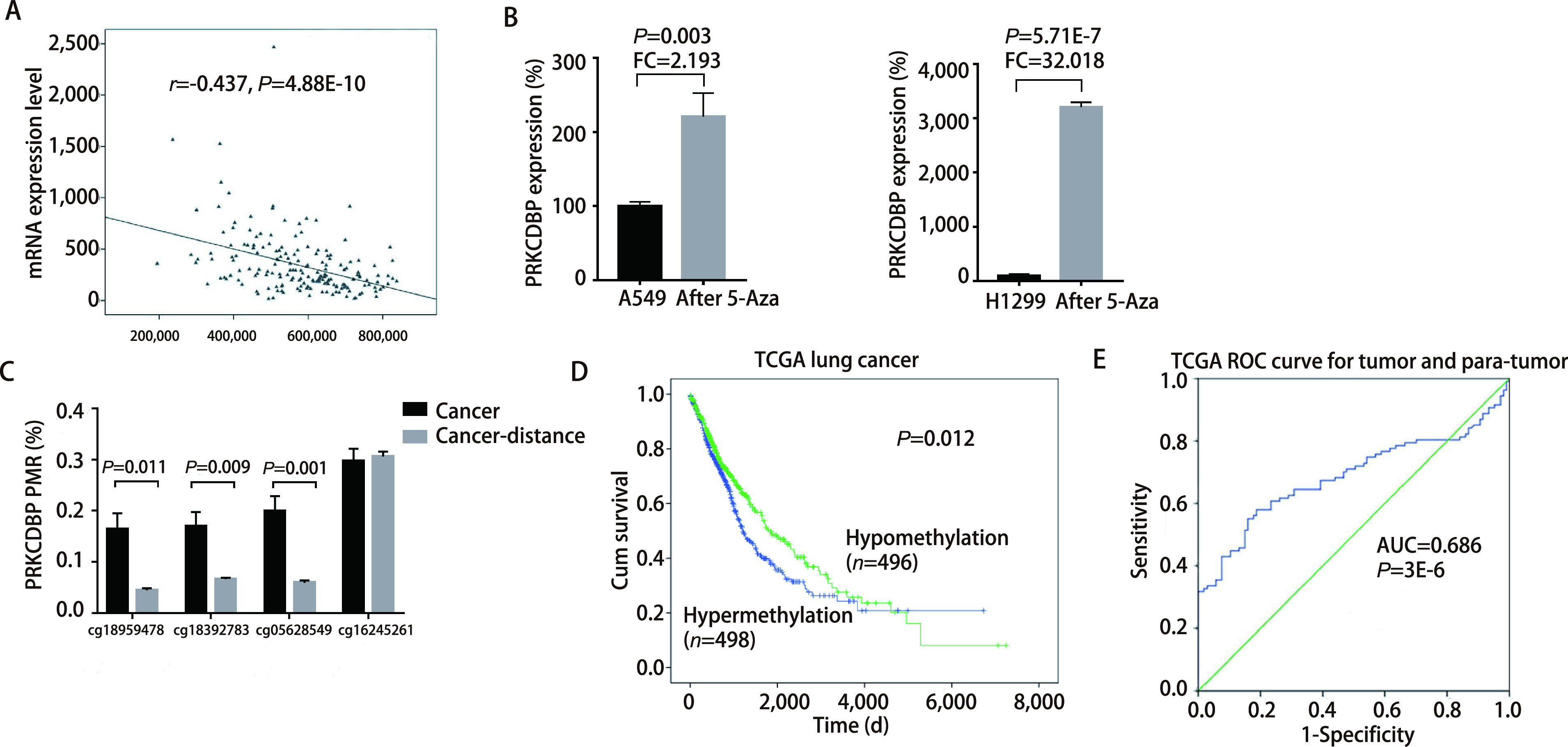
The relationship of *PRKCDBP* methylation with expression. A: The *PRKCDBP* methylation level was inversely correlated with mRNA expression in the TAGA lung cancer database (*r*=-0.437, *P*=4.88E-10); B: Changes in mRNA expression of 5'-aza-deoxycytidine treated lung cancer cell lines A549 and H1299; C: CpG methylation levels of 52 pairs of tumor tissues and para-tumor tissues in TCGA database; D: *Kaplan-Meier* analysis of the effect of differences in *PRKCDBP* methylation levels on overall survival based on TCGA data; E: Diagnostic value of *PRKCDBP* methylation for NSCLC based on the data in tumor tissues and para-tumor tissues from TCGA database.

We performed a dual luciferase assay to verify that the selected gene fragment had promoter activity. Our results showed that the transcriptional activity of the target gene plasmid pGL3-*PRKCDBP* was significantly higher than that of the pGL3-basic control plasmid, which confirmed that the fragment we analyzed could initiate gene expression [fold change (FC)=9.54, *P*=0.002]. And our results also suggest that the *in vitro* methylation of the fragment (*PRKCDBP*_Me) can significantly reduce the promoter activity of the fragment (FC=0.273; *P*=0.006, Fig 3). TCGA data analysis showed an inverse correlation between *PRKCDBP* methylation and mRNA expression (*r*=-0.437, *P*=4.88E-10; Fig 4A). In addition, we used 5'-aza-deoxycytidine demethylation on lung cancer cell lines A549 and H1299, and found that the two demethylated cell lines showed significant up-regulation of mRNA levels (A549: *P*=0.003, FC=2.193; H1299: *P*=5.713E-7, FC=32.081; Fig 4B).

## Discussion

In this study, the promoter methylation level of *PRKCDBP* was used as a potential marker for the possibly early detection of NSCLC. We found that the level of *PRKCDBP* promoter methylation was higher in tumor tissue samples, and its mRNA expression level was inversely correlated with methylation level. In the further analysis of clinical pathological data, it was found that *PRKCDBP* methylation levels of tumor tissues in all clinical pathological subtypes were higher than that in the distant non-tumor tissues. In addition, the *PRKCDBP* methylation levels between tumor tissues and para-tumor tissues were statically significant in female patients, suggesting *PRKCDBP* methylation should be a more sensitive biomarker for female NSCLC patients. These results demonstrated that *PRKCDBP* methylation should become a potential biomarker in NSCLC diagnosis.

*PRKCDBP* promoter hypermethylation have been found in colorectal^[11]^, ovarian^[13]^, gastric^[14]^, breast^[15]^ and lung cancer^[16]^. Our qMSP study provided a quantitative method which is more accurate than MSP applied in the previous studies^[17]^ in the detection of the *PRKCDBP* methylation level. *PRKCDBP* methylation was inversely correlated with mRNA expression in NSCLC patients in TCGA dataset. We also treated lung cancer cell lines (A549 and H1299) with DNA demethylation reagent 5'-aza-deoxycytidine and found the mRNA expression of *PRKCDBP* increased after treatment. These results indicated the expression of gene *PRKCDBP* was regulated by promoter methylation. We then performed double luciferase assays to determine the promoter activity of the fragment analyzed in this study and confirmed this fragment had transcriptional promoting function.

Cavin-3 (*PRKCDBP* encoding protein) is a member of the Cavin family, which is involved in the formation and functions of caveolae, including signal transduction, lipid regulation, endocytosis, and tumorigenesis^[18]^. The Cavin family gene expression is down-regulated in breast cancer. In studies of neuroblastoma^[19]^ and breast cancer^[15]^, it was found that *PRKCDBP* methylation can determine its prognosis. Our study found that the promoter of *PRKCDBP* was hypermethylated in lung cancer tissue as well as in para-tumor tissues, while was hypomethylated in the distant non-tumor tissues. It suggested that the methylation level of *PRKCDBP* might affect the development of NSCLC and the methylation changes might be detected at the early stage of its onset.

The incidence of lung cancer in China is higher in male patients than in female patients. In recent years, due to changes in smoking habits, the incidence of lung cancer in women has also shown a growing trend^[20]^. It was reported that PRKCDBP could interact with BRCA1 protein and be involved in DNA damage response and participate in the BRCA1-mediated tumor suppressor pathway^[21]^. Due to the limited sample size, we did not verify the relationship between PRKCDBP and BRCA1. However, the methylation levels of PRKCDBP promoterin female patients is higher in both tumor tissues and para-tumor tissues than in distant non-tumor tissues. It seems that PRKCDBP hypermethylation should be more sensitive in female NSCLC patients than males. This phenomenon might be related to the biologic property of PRKCDBP protein interacting with BRCA1^[10]^.

*PRKCDBP* was a downstream target of TNF α-induced tumor cell apoptosis signaling, and was activated by NF-κB^[12]^. It was also found that *PRKCDBP* could enhance the protein stability of P53, and promote apoptosis by enhancing P53 function. The hypermethylation of *PRKCDBP* causes down-regulation of P53, which promotes the progression of malignant tumors^[14]^. This also suggests a multifaceted function of *PRKCDBP* in NSCLC.

In summary, this study showed that the level of *PRKCDBP* methylation is indeed associated with NSCLC. *PRKCDBP* methylation is a potential and promising candidate biomarker for non-small cell lung cancer. The mechanism of *PRKCDBP* on NSCLC remains unclear and further experimental verification is needed in the future.
